# Pelvic heterotopic ossification causing bladder compression: a rare post-traumatic case report

**DOI:** 10.1097/RC9.0000000000000822

**Published:** 2026-08-10

**Authors:** Rose Wazwaz, Omair Bseiso, Aseel Qawasmeh, Anas Zahdeh, Wasim Aljuneidi

**Affiliations:** a Faculty of Medicine, Hebron University, Hebron, Palestine; b Orthopedic Surgeon, Almezan Hospital, Hebron, Palestine

**Keywords:** bladder compression, case report, heterotopic ossification, myositis ossificans traumatica, pelvic trauma, SCARE 2025

## Abstract

**Introduction:**

Heterotopic ossification (HO) is the pathological formation of bone within soft tissues, most commonly following trauma or surgery. Pelvic HO that causes direct mechanical compression of the urinary bladder is exceptionally rare. We present a case that remained asymptomatic for over a decade before causing significant lower urinary tract symptoms.

**Case presentation:**

A 30-year-old man presented with a 4-month history of dysuria, gross hematuria, and left testicular pain, more than 10 years after a high-energy pelvic fracture. Multimodal imaging revealed an exophytic ossified mass arising from the left pubic ramus and compressing the lateral bladder wall. Histopathology confirmed benign submucosal ossification. Surgical excision achieved complete symptom resolution by postoperative day 10.

**Clinical discussion:**

This case illustrates the capacity of traumatic HO to remain clinically silent for years before causing organ compression, with radiological overlap among neoplastic and inflammatory conditions complicating diagnosis. A multidisciplinary orthopedic–urology approach is essential.

**Conclusion:**

Pelvic HO with bladder compression should be included in the differential diagnosis of bladder masses in patients with a remote history of pelvic trauma. Early recognition and surgical excision restore urinary function.

## Introduction

Heterotopic ossification (HO) is the ectopic formation of lamellar bone within soft tissues that do not normally ossify, most commonly following musculoskeletal trauma, central nervous system injury, burns, or major orthopedic surgery^[^1,2^]^. Pelvic HO causing compression of adjacent visceral structures such as the urinary bladder is rare and diagnostically challenging.

HIGHLIGHTS

- Pelvic heterotopic ossification can cause bladder compression years after trauma.
- A careful history, imaging, and biopsy are essential for a correct diagnosis.
- Surgery can relieve urinary symptoms and restore bladder function.
- This rare case highlights the importance of including HO in the differential diagnosis.

HO must be distinguished from the broader spectrum of pathological calcifications occurring throughout the human body. Dystrophic calcification, the most common form, represents deposition of calcium salts in damaged or necrotic tissue independently of serum calcium and phosphorus levels and is typically observed in atherosclerotic plaques, healed granulomas, and necrotic neoplasms. Metastatic calcification, by contrast, results from systemic mineral imbalance, as in chronic kidney disease, hyperparathyroidism, and hypervitaminosis D, and deposits occur in otherwise healthy tissues. Calcific tendinopathy, classically affecting the rotator cuff, comprises hydroxyapatite crystal deposition within tendons through a multiphase cycle of pre-calcific, calcific (formative, resting, and resorptive), and post-calcific stages^[^3^]^. Unlike these amorphous deposits, HO is characterized by mature, organized lamellar bone with marrow elements, driven by aberrant osteogenic differentiation of mesenchymal progenitor cells^[^4^]^. Although HO is often used interchangeably with myositis ossificans, the two entities differ molecularly, with BMP-2 acting downstream of prostaglandin E2 signaling as a critical mediator of ectopic osteogenesis^[^5^]^.

HO is more prevalent in males and is associated with high-energy pelvic trauma, spinal cord injury, traumatic brain injury, and major hip surgery^[^6,7^]^. Reports of pelvic HO causing visceral compression remain scarce. This case report was prepared according to the SCARE 2025 criteria^[^8^]^.

## Case presentation

**Figure 1.**
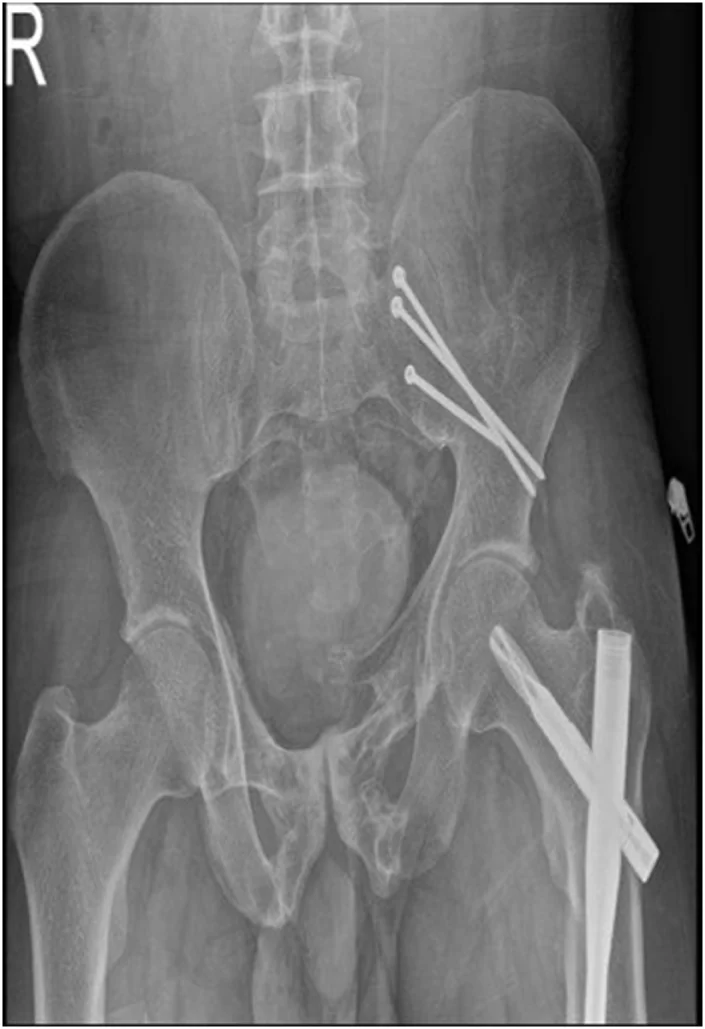
An anteroposterior plain radiograph of the pelvis and left hip demonstrating cephalomedullary (gamma) nail fixation of the left femoral neck and shaft with iliac stabilization screws, together with post-traumatic sclerotic changes in the left ischiopubic region, consistent with mature heterotopic ossification.

A 30-year-old male presented with a 4-month history of dysuria (worse with a full bladder and at the end of micturition), gross hematuria, and left testicular pain. Approximately 10 years earlier, at 20 years of age, he had sustained polytrauma in a high-energy motor vehicle collision, comprising an unstable anteroposterior compression–type pelvic ring injury with disruption of the anterior pelvic brim (left superior and inferior pubic ramus fractures with symphyseal diastasis), a left femoral neck and shaft fracture, an extraperitoneal bladder laceration of the dome and left lateral wall, and a partial bulbar urethral disruption. Initial management followed a staged trauma protocol: emergency resuscitation and external pelvic fixation, followed by definitive open reduction and internal fixation. The femoral fractures were stabilized with a cephalomedullary (gamma) nail; the pelvic ring was reinforced with bilateral iliac bone screws and a reconstruction plate across the symphysis pubis; the bladder laceration was repaired primarily in two layers via a transabdominal approach, with concurrent urethral Foley and suprapubic catheter drainage; and the bulbar urethral injury was managed by primary realignment over a Foley catheter. He subsequently developed a bulbar urethral stricture that was treated by urethral dilation and optical urethrotomy, after which he remained asymptomatic for nearly a decade. The retained hardware is shown in Figure 1. The full chronological sequence is summarized in Table 1.

**Table 1:** Clinical timeline.

Approximate date	Event	Details
~2013–2014	Initial trauma	High-energy motor vehicle collision; unstable pelvic fracture, bladder injury, urethral injury. Managed surgically with cephalomedullary (gamma) nail fixation of left femur, pelvic stabilization with iliac screws, and bladder repair.
~2014–2015	Urological complication	Patient developed dysuria. Retrograde urethrogram revealed bulbar urethral stricture. Managed with urethral dilation followed by optical urethrotomy.
~2016	Urological reassessment	Pre-sphincter urethral narrowing without functional impairment. Mildly reduced flow on uroflowmetry. Asymptomatic thereafter.
2014–2023	Asymptomatic period (~10 years)	No urinary or pelvic complaints. No interval imaging. HO developing silently in pelvic soft tissues.
~Early 2024	Symptom onset	Insidious onset of dysuria, gross hematuria, and left testicular pain.
Late 2024/Early 2025	Diagnostic workup	Scrotal Doppler, abdominal ultrasound, CT pelvis, pelvic MRI, cystoscopy, and bladder biopsy. Diagnosis of pelvic HO established.
Early 2025	Surgical intervention	Retroperitoneal excision; combined orthopedic-urology team. Intraoperative bladder injury managed. Foley catheter and antibiotic prophylaxis.
Day 10 post-op	Follow-up	All symptoms resolved. Foley catheter removed. Patient voided without difficulty.

On examination, the patient was hemodynamically stable, with mild suprapubic tenderness on deep palpation and a left-sided varicocele, confirmed with the Valsalva maneuver. The neurological and remaining systemic examinations were unremarkable. Laboratory tests (CBC, CMP, ESR, CRP, serum calcium, phosphorus, and alkaline phosphatase) were within normal limits. Urinalysis showed microscopic hematuria with a negative culture.

**Figure 2.**
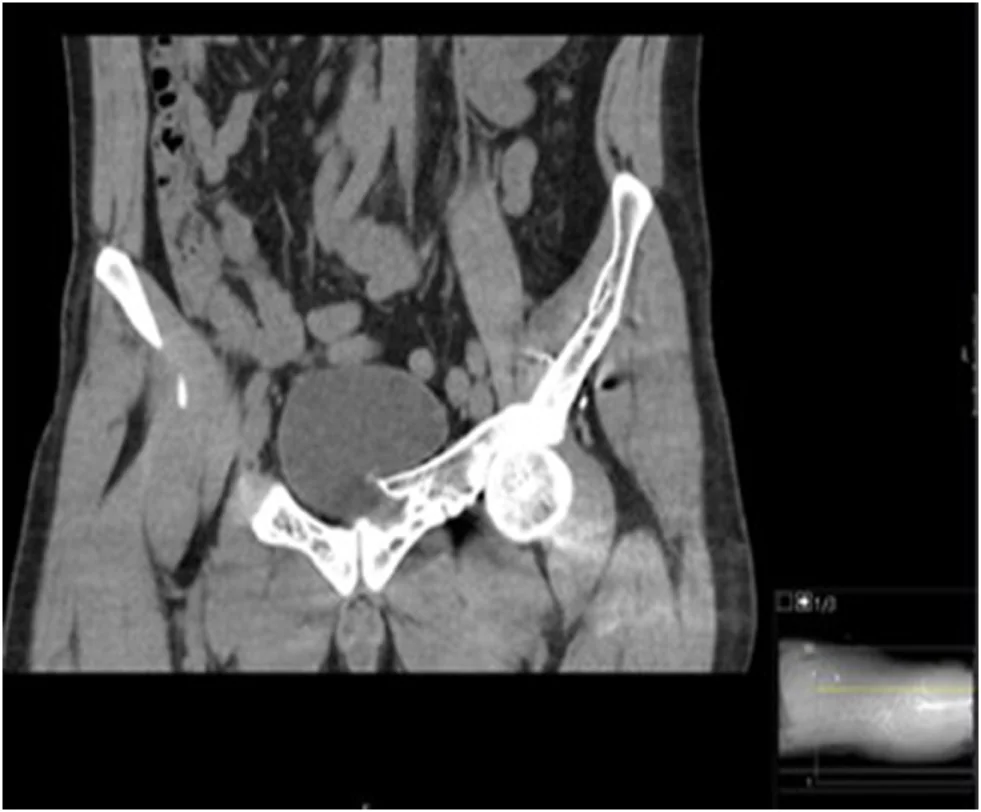
Pelvic CT (coronal reformation) demonstrates mature sclerotic heterotopic ossification arising from the left ischium and extending toward the urinary bladder, with retained cephalomedullary (gamma) nail and iliac stabilization screws from prior pelvic reconstruction.

**Figure 3.**
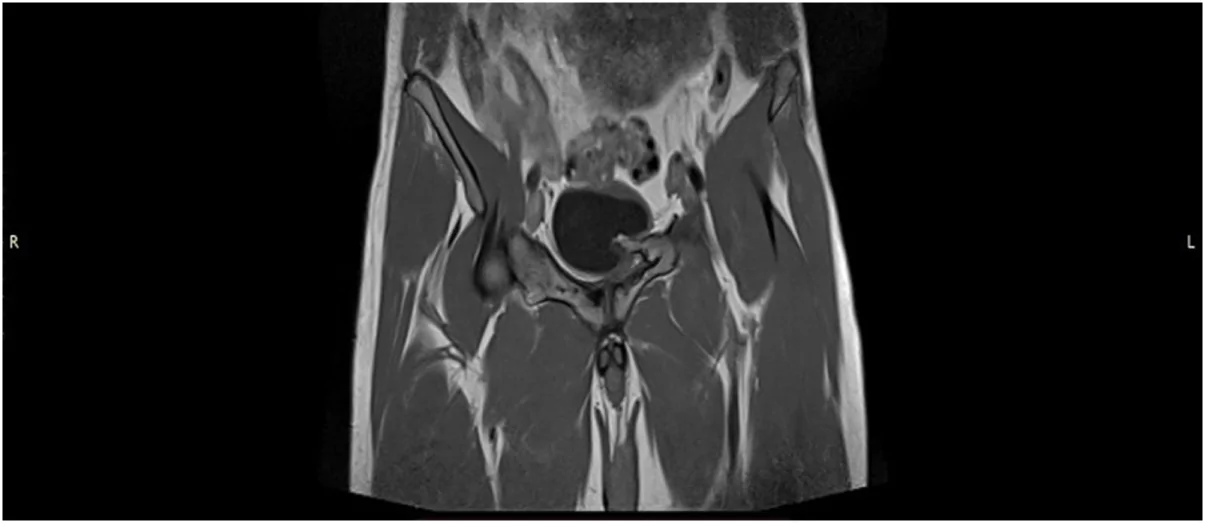
Pelvic MRI (T2-weighted coronal sequence) demonstrating a 1.7-cm exophytic bony protuberance arising from the upper cortex of the left pubic ramus, abutting and compressing the left lateral bladder wall, with adjacent reactive wall thickening, consistent with an inflammatory response to chronic mechanical compression.

Cystoscopy demonstrated an extrinsic impression on the left lateral bladder wall without mucosal ulceration or intraluminal mass. Cold-cup biopsy of the area of wall thickening revealed benign urothelial mucosa with focal submucosal ossification, excluding dysplasia and malignancy. Abdominal ultrasound showed an ill-defined echogenic shadow at the inferior aspect of the left lateral bladder wall (1.9 × 1.5 cm) with posterior acoustic shadowing. CT of the pelvis (120 kVp, 250–350 mAs, 3 mm slices) confirmed mature sclerotic ossification arising from the left ischium and extending toward the bladder, with a retained cephalomedullary nail and iliac screws (Fig. 2). Pelvic MRI (1.5 T; T1, T2, STIR, post-contrast T1 fat-saturated) showed an exophytic 1.7 cm bony protuberance on the upper cortex of the left pubic ramus, abutting the left lateral bladder wall with adjacent inflammatory wall thickening; no enhancing intraluminal lesion was identified (Fig. 3). Scrotal Doppler showed a grade II left varicocele (Sarteschi), accounting for testicular pain. Key differentials are summarized in Table 2.

**Table 2:** Differential diagnoses.

Differential diagnosis	Key features	Diagnostic tests
Heterotopic ossification (HO)	Remote trauma history; mature lamellar bone outside skeleton on imaging; biopsy benign; extrinsic bladder compression	X-ray, CT, MRI, histopathological biopsy
Urothelial carcinoma	Intraluminal mass; mucosal irregularity; hematuria; risk factors (smoking); biopsy dysplasia/malignancy	Cystoscopy with biopsy, CT urography, MRI contrast
Pelvic bone tumor/osteochondroma	Bony exostosis; extrinsic mass effect; may lack trauma history	CT, MRI, histopathology
Post-traumatic fibro-osseous scar	Trauma/surgery history; mixed fibrous-ossific components; less organized than mature HO	CT, MRI
Bladder endometriosis	Reproductive-age women; cyclic hematuria; extrinsic or intramural lesion	MRI, laparoscopy, biopsy
Urethral stricture	Reduced flow; urethral injury history	Retrograde urethrogram, uroflowmetry
Varicocele (testicular pain)	Soft scrotal mass; bag-of-worms; no bladder mass	Scrotal Doppler

Surgical excision was performed under general anesthesia via a retroperitoneal Pfannenstiel approach, with a urologist present from the outset. The HO appeared as a firm, whitish, chalky mass with disorganized trabecular architecture, distinguishing it from native bone, and was densely adherent to the left lateral bladder wall. Dissection was performed using periosteal elevators, a high-speed burr, osteotomes, and monopolar and bipolar electrocautery for hemostasis. During mobilization, a 1.5-cm cystotomy occurred at the thinned and fibrotic bladder wall, which was classified as an AAST grade II laceration. The urologist performed an immediate two-layer primary repair using 2-0 polyglycolic acid (Vicryl) sutures: a running inner mucosal–submucosal layer followed by an interrupted outer seromuscular layer. Watertight integrity was confirmed by intraoperative bladder distension with 200 mL of methylene-blue-tinted saline, which demonstrated no extravasation. The repair was then buttressed with mobilized omentum to provide vascularized coverage and interpose viable tissue between the suture line and the excision bed. A 16-Fr urethral Foley catheter and a 14-Fr suprapubic cystostomy were placed for dual bladder decompression, and a closed-suction pelvic drain was positioned in the perivesical space.

Postoperative bladder management followed AUA Urotrauma Guideline principles^[^9^]^. Continuous bladder decompression was maintained via both catheters for 10 days, with daily monitoring of catheter output. Antibiotic prophylaxis comprised IV cefazolin 1 g every 8 hours for 48 hours, followed by oral ciprofloxacin 500 mg twice daily for 8 days. The pelvic drain was removed on day 3 following the cessation of significant output. Urinalysis and urine culture on days 3 and 7 were negative. A confirmatory cystogram was performed on day 9, demonstrating an intact bladder without extravasation; the suprapubic catheter was clamped, a successful trial-of-void was completed, and both catheters were removed on day 10. No NSAID or radiotherapy prophylaxis was administered, given the mature, quiescent histology, complete excision, 10-year interval since the original injury, and proximity to the bladder and genitalia.

At the 10-day follow-up, the patient reported complete resolution of dysuria, gross hematuria, and testicular pain, with spontaneous voiding and a normal urinalysis. Follow-up CT/MRI is planned at 3 and 6 months post-excision to assess HO recurrence and bladder-wall remodeling.

## Discussion

Pelvic HO with bladder compression is exceptionally rare. The most directly comparable precedent is the case reported by McMahon *et al*, in which retroperitoneal HO progressively encased the ureter following spinal cord injury, ultimately necessitating nephrectomy^[^10^]^. The present case differs in the longer asymptomatic interval (≥10 years), the distinct anatomical target (bladder rather than ureter), and successful organ-preserving surgical management. Differential diagnoses are summarized in Table 2.

The diagnostic pathway was structured around the indeterminate ultrasound finding: cystoscopy and biopsy were performed before advanced cross-sectional imaging to exclude urothelial malignancy. The histological diagnosis of submucosal ossification then redirected the investigations toward characterization of the underlying bony lesion. CT proved superior for assessing ossification maturity and cortical definition (Fig. 2), while contrast-enhanced MRI defined soft-tissue involvement and excluded an enhancing mucosal lesion (Fig. 3), in keeping with published evidence^[^11^]^.

The pathophysiology of traumatic pelvic HO involves aberrant osteogenic differentiation of mesenchymal progenitors within an inflammatory and hypoxic post-traumatic environment, which distinguishes this process from the amorphous deposits seen in dystrophic and metastatic calcification. Spicule-type anterior pelvic HO is more common in males after high-energy fractures with visceral involvement and metallic hardware^[^12^]^. BMP-2, downstream of prostaglandin E2 signaling, is a key molecular mediator^[^5^]^. A clinically silent interval of a decade, as observed here, is consistent with the slow incremental maturation of HO before it reaches a size sufficient to compress adjacent organs.

The intraoperative cystotomy reflects the hazards of dissection in chronic perivesical HO: chronic compression and reactive fibrosis had thinned the bladder wall and caused it to adhere to the ossification interface. Two-layer primary repair with omental buttressing and dual urethral–suprapubic drainage are well-established techniques for traumatic and iatrogenic bladder injury, with current guidelines recommending confirmatory cystography before catheter removal^[^9^]^. Pharmacological prophylaxis (NSAIDs) and radiotherapy prophylaxis are well supported for the early post-injury period. A systematic review of more than 5000 patients undergoing acetabular fixation showed that NSAIDs reduced HO from 36% to 27%, and that radiotherapy reduced severe ossification to approximately 3%^[^13^]^. However, these data do not extend to mature, decade-old quiescent lesions. The testicular pain was attributable to the coexisting varicocele rather than HO, a reminder that converging symptoms in young males may have distinct etiologies.

### Limitations

The single-case design limits generalizability. Follow-up at ten days is too short to assess HO recurrence; planned 3- and 6-month imaging will address this. Intraoperative photographic documentation was not obtained during the urgent management of the cystotomy.

## Conclusion

Pelvic HO with bladder compression is a rare delayed complication of high-energy pelvic trauma that may remain silent for over a decade. CT and contrast-enhanced MRI, combined with histopathology, enable an accurate diagnosis, and a multidisciplinary excision can achieve sustained symptom resolution while preserving bladder integrity. HO should be included in the differential diagnosis of any bladder mass in patients with a remote history of pelvic trauma.

## Data Availability

All data relevant to this case report are included within the published article. No additional datasets were generated or analyzed for this study.

## SCARE checklist

Prepared in accordance with the SCARE 2025 guidelines.
